# Cardiovascular Risk in Systemic Autoimmune Diseases: Epigenetic Mechanisms of Immune Regulatory Functions

**DOI:** 10.1155/2012/974648

**Published:** 2011-09-14

**Authors:** Chary López-Pedrera, Carlos Pérez-Sánchez, Manuel Ramos-Casals, Monica Santos-Gonzalez, Antonio Rodriguez-Ariza, Ma José Cuadrado

**Affiliations:** ^1^Unidad de Investigación e Instituto Maimónides de Investigación Biomédica de Córdoba (IMIBIC), Hospital Universitario Reina Sofía, Avda. Menéndez Pidal s/n, 14004 Córdoba, Spain; ^2^Laboratory of Autoimmune Diseases Josep Font, IDIBAPS, Department of Autoimmune Diseases, Hospital Clínic, C/ Villarroel 170, 08036 Barcelona, Spain; ^3^Lupus Research Unit, St Thomas Hospital, Lambeth Palace Road, London SE1 7EH, UK

## Abstract

Autoimmune diseases (AIDs) have been associated with accelerated atherosclerosis (AT) leading to increased cardio- and cerebrovascular disease risk. Traditional risk factors, as well as systemic inflammation mediators, including cytokines, chemokines, proteases, autoantibodies, adhesion receptors, and others, have been implicated in the development of these vascular pathologies. Yet, the characteristics of vasculopathies may significantly differ depending on the underlying disease. In recent years, many new genes and signalling pathways involved in autoimmunity with often overlapping patterns between different disease entities have been further detected. Epigenetics, the control of gene packaging and expression independent of alterations in the DNA sequence, is providing new directions linking genetics and environmental factors. Epigenetic regulatory mechanisms comprise DNA methylation, histone modifications, and microRNA activity, all of which act upon gene and protein expression levels. Recent findings have contributed to our understanding of how epigenetic modifications could influence AID development, not only showing differences between AID patients and healthy controls, but also showing how one disease differs from another and even how the expression of key proteins involved in the development of each disease is regulated.

## 1. Introduction

Autoimmune diseases are a heterogeneous group of disorders characterised by humoral, cell-mediated immune responses against various self-constituents. It is widely known that AIDs are the result of interaction between predisposing genetic factors, deregulation of the immune system, and environmental triggering factors [1]. Several systemic autoimmune conditions, including rheumatoid arthritis (RA), systemic lupus erythematosus (SLE), antiphospholipid syndrome (APS), and primary Sjögren Syndrome (pSS) are linked to enhanced atherosclerosis, and consequently higher cardiovascular morbidity and mortality rates. The development of cardiovascular disease involves genetic factors as well as other acquired and modifiable risk factors (e.g., hypercholesterolemia, diabetes mellitus, and hypertension). Inflammatory components of the immune response, as well as autoimmune elements (e.g., autoantibodies, autoantigens, and autoreactive lymphocytes), seem to be also involved in these processes [2–6]. 

From the genetic standpoint, it is known that in the case of AID there is a complex interaction between the product of various genes, and genomic high-throughput analyses can tell us which genes are turned on or off in different tissues from patients with autoimmune diseases. Recent genomic and transcriptomic profiling studies have implicated certain cytokines, surface receptors, signalling pathways, and cell types in the pathogenesis of inflammatory diseases [7–17]. This paper is focused in epigenomic approaches used to deep into the origin of the mechanisms associated with both disease development and vascular involvement in systemic autoimmune diseases.

## 2. Epigenetic Mechanisms in Autoimmune Diseases

Systemic autoimmune diseases (AIDs) are of complex aetiology, characterised by an intricate interplay of various factors. A myriad of genes lies behind the heterogeneous manifestations of these diseases, and the overexpression and repression of particular genes form a specific gene expression profile (GEP) (genetic fingerprints) that is characteristic to the given disease phenotype. Pathophysiological mechanisms that might connect atherosclerosis and cardiovascular disease with SLE and RA have been greatly broadened with the application of genomic technologies, which have allowed explaining how these alterations might be associated to each AID [7–10, 12, 13]. One important and emerging mechanism controlling gene expression is epigenetics.

Epigenetics, the control of gene packaging and expression independent of alterations in the DNA sequence, is providing new directions linking genomics and environmental factors. The epigenetic process is important for controlling patterns of gene expression during the cell cycle, development, and in response to environmental or biological modifications. Moreover, epigenetic changes may be reversed [18].

A remarkable example of disease in which epigenetic abnormalities and patterns of inheritance are extremely complex is SLE. The high incidence of twin pairs in which SLE develops in only one of the siblings supports the notion that environmental factors and their involvement in epigenetic modifications could affect the onset of disease. 

**Figure 1:**
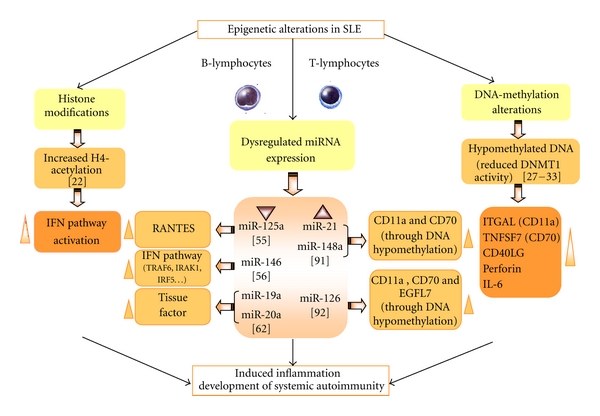
Epigenetics alterations in SLE and potential pathogenic contributions to inflammation, CVD, and autoimmunity. See the text for further details.

**Figure 2:**
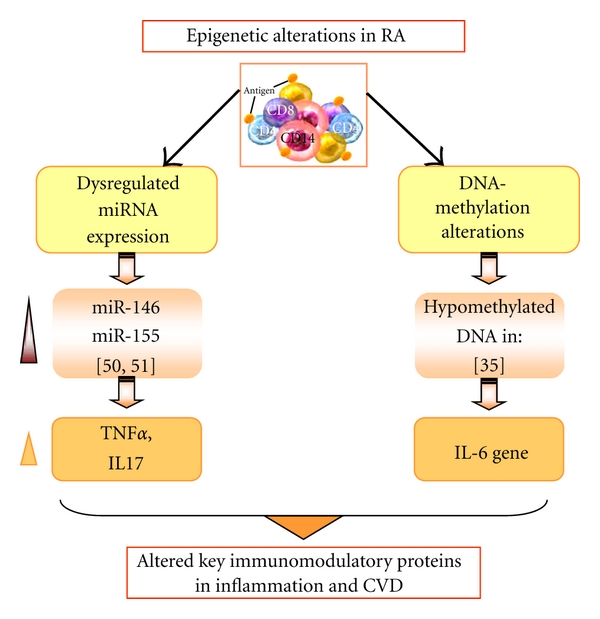
Epigenetic alterations in RA and potential pathogenic contributions to inflammation and CVD. See the text for further details.

In the last year, several new findings about epigenetic modifications of gene expression were reported in different AIDs. These modifications describe changes in the expression of DNA that result from methylation, posttranslational modifications of the histone proteins, including acetylation/deacetylation, methylation, and microRNAs (Figures 1 and 2). Most interestingly, these modifications seem to act in concert [19]. 

### 2.1. Histone Modifications

Histone modifications are regulated during the cell cycle, cellular development, and differentiation [20]. The two main histone modifications, histone acetylation and histone methylation, are tightly controlled. Indeed, histone acetylation is counterbalanced by histone deacetylation: histone acetyl transferase (HAT) adds the acetyl group, and the histone deacetylases (HDAC) remove it. HATs and HDACs reciprocally regulate the acetylation status of cellular proteins. Acetylation of histones promotes unwinding of compacted chromatin and allows access of transcription factors to gene promoter regions, while deacetylation of terminal lysine residues contributes to the silencing of transcription. Changes in relative HAT/HDAC activity would influence the sensitivity of cellular gene transcription in response to extracellular stimuli.

Similarly, the impact of lysine or arginine methylation by histone methyltransferases (HMTs) is reversed by demethylating enzymes, such as lysine-specific demethylase (LSD)1 and JmjC domain-containing histone demethylase (JMJC).

Global H3 and H4 hypoacetylation and hypermethylation characterize CD4+ T cells from SLE patients [21]. Moreover, a very recent study has demonstrated that there are significant clusters of aberrantly expressed genes in SLE (including those codifying for a set of chemokines) which are strongly associated with altered H4 acetylation [22].

### 2.2. DNA Methylation Alterations

DNA methylation occurs by covalent addition of a methyl group from the methyl donor S-adenosylmethionine (SAM) to the 5′ carbon of the cytosine ring in CpG pairs. Throughout the genome CpG is often found clustered in particular regions called CpG islands. CpG islands are typically methylated in silenced genes and hypomethylated in the regulatory areas of transcriptionally active genes. DNA methyl addition is carried out by at least five DNA methyl transferases (DNMT1, DNMT3a, DNMT3b, DNMT3L, and DNMT2). DNMT1 contributes to the maintenance of DNA methylation patterns, while DNMT3a and -b methylate unmethylated DNA and thus contribute to the novo methylation. DNMT2 displays weak DNA methyl transferase activity [18].

It has been shown that hydralazine and procainamide remove the methyl group from cytosines present in CpG islands through their interaction with DNA [23]. 

Procainamide and 5-azacythidine are competitive inhibitors of DNMT1 [24, 25], while hydralazine prevents DNMT1 upregulation during mitosis by blocking ERK pathway signaling at PKCdelta [26]. 

Patients with idiopathic lupus have changes in T cell signaling identical to those caused by hydralazine. In fact it has been demonstrated that T cells from patients with active lupus have hypomethylated DNA, due to decreased DNMT1 levels and activity [27, 28]. Interestingly, the decrease in DNMT1 levels is due to impaired ERK pathway signaling caused by a block at PKCdelta, also inhibited by hydralazine [26]. 

Candidate gene studies have revealed several pathways in which aberrant gene expression due to DNA demethylation is linked with the development of SLE. These genes include the ITGAL (also known as CD11A) [29], which is important for cell-cell adhesion, CD70 (encoding CD70), (also known as tumor necrosis factor ligand super family member 7) [30], which is required for T cell proliferation, clonal expansion, and the promotion of effector T cell formation, and CD40LG (encoding for CD40 ligand) [31], which stimulates B cell IgG overproduction. Other factors, such as the gene encoding perforin 1 (PRF1), [32] which contributes to autoreactive killing of macrophages and release of apoptotic material, are also hypomethylated in CD4+ T cells from individuals with SLE.

In 2009, Garaud and colleagues [33] reported that the E1B promoter of CD5 is hypomethylated in resting SLE B cells. This study also showed that high levels of interleukin 6 in SLE B cells, which is known to be positively associated with SLE disease activity, reduce the expression of DNMT1.

Defective DNA methylation was also described in RA. T cell DNA is demethylated in RA [34] and may result in the generation of auto reactive T and/or B cell clones in RA as it does in lupus. The CD21 promoter is also demethylated in RA PBMC and synovial fluid cells. IL-6 has been further shown to be hypomethylated in PBMCs from individuals with RA [35]. However, further analysis of isolated B cells is necessary to confirm whether deregulated IL-6 expression occurs in these cells as a consequence of epigenetic changes in RA. Nevertheless, altered methylation of IL6 in RA reinforces the notion of the importance of applying epigenetic studies to the investigation of pathways that are affected in AIDs.

In pSS, recent studies have focused on analyzing DNA methylation alterations in mechanotransduction and hemidesmosome (HD) organization-mediated mechanisms. Changes in cell behaviour depend on mechanical signals received by the cell from the environment (mechanotransduction) [36, 37]. Experimental evidence suggests that a physical continuum directly connects the extracellular matrix (ECM) to the cellular nucleus [37]. Recent studies have investigated the role of these new mechanisms in the pathogenesis of SS and, specifically, the role of epigenetic processes in the development of glandular damage. An example of a mechanotransduction-mediated mechanism is the production of lactotransferrin by glandular cells: high levels of mRNA for lactotransferrin have been detected in a cell fraction enriched in epithelial cells from salivary glands of patients with SS, together with an altered distribution of *α*6*β*4 integrin and an acinar cell shape [38]. Increased transcription of the lactotransferrin gene suggests a role of mechanotransduction-signalling pathways in the etiopathogenesis of SS. 

A recent study by González et al. [39] found alterations in type I HD components in the salivary glands of SS patients suggestive of epigenetic control. HDs are protein complexes that mediate epithelial cell adhesion to the ECM and are composed of an *α*6*β*4 integrin dimer that binds to laminin, plectin, and other proteins (BP230 and BP180) [40, 41]. The study found reduced levels of BP230 mRNA in epithelial cells of patients with SS in comparison with controls and, in contrast, an accumulation of BP230 on the basal surface of acini [39]. An increased methylation index of CpG islands might explain the reduced levels of BP230 mRNA, and the authors suggest that differential changes in methylation of the BP230 gene promoter may explain the up- and downregulation detected in patients with SS. More recently, Yin and coworkers [42] evaluated whether the epigenetic regulation of CD70 expression is abnormal in pSS. They found that CD70 expression was significantly elevated and correlated with a decrease in TNFSF7 promoter methylation in pSS CD4(+) T cells compared to controls. 

This study indicated that, as for SLE, demethylation of the CD70 promoter regulatory elements contributes to CD70 over expression in pSS CD4(+) T cells and may further contribute to auto reactivity.

### 2.3. MicroRNAs in Autoimmune Diseases

miRNAs are short (approximately 22 nucleotides in length) molecules of RNA that are transcribed from noncoding regions of the genome and exhibit significant secondary structure [43]. 

The biosynthesis of miRNAs is mediated by the nucleases Drosha, Pasha, and Dicer, involved in processing the miRNA from a hairpin configuration into a short RNA duplex and finally into a single-stranded miRNA, which is then loaded into the mRNA-induced silencing complex (RISC). The RISC and associated miRNA then bind complementary sequences in 3′ untranslated regions (base pairing of microRNA nucleotides 2–8, termed seed sequence) of mRNA species and inhibit their translation by two distinct mechanisms: degradation of the message by the RISC protein argonaute and prevention of ribosomal binding and translation initiation [43].

miRNAs play a key role in biological processes, such as embryogenesis, differentiation and proliferation of cells, production of cytokines, and apoptosis. More than 700 miRNAs have been identified in mammalian cells, and up to one-third of all protein-encoding genes are estimated to be regulated by these small molecules [44]. 

miRNA expression is tightly regulated during hematopoiesis and lymphoid cell differentiation, and disruption of the entire miRNA network of selected miRNAs may lead to dysregulated immune responses. In fact, abnormalities in miRNA expression related to inflammatory cytokines, Th-17, and regulatory T cells as well as B cells have been described in several AIDs [45].

In addition, it has been shown that miRNAs are present in human plasma in a stable form. Moreover, other body fluids such as synovial fluids contain measurable miRNAs [46]. Thus, synovial fluid and plasma miRNAs have additional potential as diagnostic biomarkers for some AIDs as well as a tool for the analysis of their pathogenesis.

#### 2.3.1. MicroRNAs in Rheumatoid Arthritis

The expression levels of several miRNAs in PBMCs have been found associated with inflammation and cytokine production, and some of them correlated with RA disease activity [47].

In reports from different research groups, miR-146a and miR155 have been consistently found to be up regulated in synovial fibroblasts (RASFs), PBMCs, synovial fluid, PBMC-derived CD4+ T cells, and Th-17 cells from patients with RA when compared with healthy controls or patients with osteoarthritis (OA) [40, 48–51].

The study of the by Li and colleagues [50] of the expression profile of miRNAs in CD4+ T cells from synovial fluid and peripheral blood of 33 RA patients showed that miR-146a expression was significantly up regulated, while miR-363 and miR-498 were down regulated in RA patients. Moreover, the level or miR-146a expression was positively correlated with levels of tumor necrosis factor-alpha (TNF*α*). In addition, miR-146a overexpression was found to suppress T cell apoptosis, thus indicating a role for miR-46a in RA pathogenesis. 

As a proof of the additional involvement of miR-146a in inflammation and cytokine production, Niimoto and coworkers [51] recently found that the expression of miR-146a was associated with IL-17 expression in the PBMC and synovium in RA patients and that the increased expression of both molecules correlated with disease activity.

A parallel survey further demonstrated that a polymorphism in the 3′-UTR of interleukin-1 receptor-associated kinase (IRAK1), a target gene of miR-146a, is associated with RA susceptibility [52].

Simultaneous investigations in PBMCs from acute coronary syndrome patients further showed significantly increased expression of miR-146a [53]. Moreover, the over expression of miR-146a was found to significantly unregulate the function of Th1 cells. This study also provided evidence that miR-146a treatment in vitro could induce the protein expression of TNF*α*, MCP-1 and NF*κ*B p65, which are, respectively, key proinflammatory cytokines and critical transcription factor in atherosclerosis. Although further detailed studies are required, these results support the hypothesis that miR-146a may be directly involved in the pathogenesis of CVD associated to AIDs such as RA.

#### 2.3.2. miRNAs in Systemic Lupus Erythematosus and Antiphospholipid Syndrome

The first study reported about miRNA expression in PBMCs from SLE was the study by Dai and coworkers [54], performed in 23 SLE patients and 10 healthy controls. In those SLE patients, 7 miRNAs (miR-196a, miR-17-5p, miR-409-3p, miR-141, miR-383, miR-112, and miR-184) were down regulated and 9 miRNAs (miR-189, miR-61, miR-78, miR-21, miR-142-3p, miR-342, miR299-3p, miR-198, and miR-298) were up-regulated as compared with healthy controls. However, that study did not provide further data to show how these changes in miRNA may play a role in SLE disease pathogenesis.

A recent study has shown the involvement of miR-125a in the inflammatory chemokine pathway in SLE [55]. In SLE patients, the expression of miR-125a was found to be reduced, and the expression of its predicted target gene, KLF13, was increased. This study also showed that miR-125a negatively regulated RANTES expression by targeting KLF13 in activated T cells. 

It has also been recently demonstrated the down regulation of miR146 in PBMCs from SLE patients, as well as its involvement in IFN over expression [56]. 

APS and SLE are two conditions known to provoke increased tissue factor (TF) expression in monocytes and endothelial cells [57–61]. In a recent study by our group, we checked in patients with APS or SLE the hypothesis that miRNA levels may influence TF levels in those patients. Thus we measured by RT-PCR the levels of miR-19b and miR-20a (reported to targeting TF expression in several web databases and algorithms of miRNA target prediction) in monocytes from APS and SLE patients. In APS we found that the levels of these two miRNAs had an approximate 3-4-fold decrease in comparison with monocytes from healthy controls. In monocytes from SLE, miR-20a levels were also lower than those from healthy subjects (3-fold decrease). In addition, the reduced expression of miR-19b and miR-20a was inversely correlated with TF cell surface expression [62]. These results shed light on new mechanisms that may regulate the expression of TF and thus the occurrence of thrombotic events in these AIDs.

#### 2.3.3. miRNAs in Primary Sjögren Syndrome

Studies on the role of microRNAs (miRNAs) in the pathogenesis of pSS have centred on analysing miRNAs from salivary exosomes. Exosomes are small cellular vesicles (30–100 nm) that contain a wide range of surface and internal proteins specific to their cellular origin [63], and recent studies have shown that exosomes can also transport mRNA and miRNA [64, 65]. Alevizos et al [66] tested the potential of salivary gland miRNAs as a biomarker of SS, using Agilent microRNA microarrays to profile miRNAs isolated from the salivary glands of healthy controls (*n* = 8) and patients with SS, who were classified according to a high focus (*n* = 8) or low focus score (*n* = 8). MicroRNA expression patterns distinguished salivary glands from control subjects and the two groups of patients with SS. The authors identified two miRNAs (768-3p and 574) which were inversely correlated with the focus score. In addition, they found down-regulation of the mir-17-92 cluster in half the SS patients with a high focus score. Previous studies have associated down-regulation of the mir-17-92 cluster with an accumulation in pro-B cells and a marked reduction of pre-B cells, which has been associated with lymphoproliferative and autoimmune diseases [67, 68]. Larger studies are currently underway to validate miRNAs from salivary glands as diagnostic markers in SS [69]. Moreover, parallel analyses on PBMCs from both mice and human are currently underway so that Lu and co-workers [70] have presented preliminary data describing two miRNAs (150 and 146) that are up regulated in both, target tissues and in PBMCs of the B6DC mice, and in PBMCs and salivary glands of SS patients.

## 3. Epigenetic Alterations, Inflammation, and Cardiovascular Involvement in Autoimmune Diseases

Significant evidence has shown that there is heterogeneity in the characteristics of vasculopathies underlying different autoimmune diseases such as APS, SLE, RA, and pSS. It has been also shown a relevant heterogeneity with respect to inflammatory risk factors. The data presented in this revision further indicated that epigenetic mechanisms also seem to influence inflammation and cardiovascular disease in those autoimmune conditions. 

In SLE, relevant factors directly influencing the development of CVD and AT comprise immune complex generation, complement activation, and changes in the production and activity of a complex network of cytokines, including type I and II interferons, B lymphocyte stimulator (BLyS), TNF*α*, IL-6, IL-17, and migration macrophage inhibitor (MIF) [71–82]. Epigenetic analyses have demonstrated aberrant gene expression due to DNA methylation linked to both, the development of the disease and to inflammation and AT (such as ITGAL, CD70, and CD40L) [28–30]. There have been also found significant clusters of aberrantly expressed genes in SLE (codifying for a set of chemokines) strongly associated with altered H4 acetylation [22]. Concerning altered miRNA expression, various studies have demonstrated in PBMCs altered expression of some miRNAs (miR-125a and miR-146) involved in the regulation of inflammatory chemokine pathways as well as of IFN over expression [54, 55].

In Antiphospholipid Syndrome (APS), an autoimmune disease in which thrombosis development constitutes a major pathological feature, procoagulant cell activation, accompanied with TF expression and TF pathway up regulation, is one of the key events considered explaining the prothrombotic tendency [57–61]. Although this pathology has been also associated with inflammatory condition and early AT development, no epigenetic studies have been developed to date to prove any relationship with those pathogenic processes. Nevertheless, along with SLE, the over expression of TF has been demonstrated in APS to be accompanied with an epigenetic change: the altered expression of miR-19b and miR-20a [62]. Although more deep studies are required, this association reveals a new mechanism involved in the pathophysiology of thrombosis in both autoimmune diseases.

In RA, pathogenic mechanisms involved in CVD and AT development include prooxidative dyslipidemia, insulin resistance, prothrombotic state, and immune mechanisms such as T cell activation that subsequently leads to endothelial dysfunction and arterial stiffness [83, 84]. Anticyclic citrullinated peptide antibodies (anti-CCP), IgM rheumatoid factor, circulating immune complexes, proinflammatory cytokines including TNF*α* and IL-6, Th0/Th1 cells, decreased folate and vitamin B12 productions, and impaired paraoxonase activity, among others, may all be further involved in the development of vascular disease in RA [85–87]. Epigenetic data indicate that defective DNA methylation might also be relevant to CVD and AT development in RA, so that it has been shown hypomethylation of IL-6 promoter [35]. Furthermore, the expression levels of some miRNAs in PBMCs have been found associated with inflammation and cytokine production, including the over expression of miR-146a (which positively correlated with levels of TNF*α* and IL-17) [50, 51].

In primary SS, various studies concluded that there is evidence that those patients have both early subclinical AT and altered lipid profile with potential AT risk. In that pathology, the key role in determining the acceleration of AT seems to be played by immune-mediated mechanisms. Numerous interferon regulatory genes have been also found to be highly expressed in T cells and in salivary gland tissue [14–17].

Concerning epigenetic studies suggesting inflammation and CVD involvement in SS, to date only one group have reached concluding results. The study group of Cha and co-workers has developed non obese diabetic mice (B6DC) that develop a disease similar to human SS. They have described two miRNAS (150 and 146) that are up regulated in target tissues and in PBMCs of the B6DC mice compared to control mice. That group further reported that miR-146 expression is increased in PBMCs and salivary glands of SS patients [70], which might be related to IFN production. Hypomethylation and over expression of CD70 (TNFSF7) in CD4+ T cells of patients with primary Sjögren's syndrome have been also demonstrated [42]. Although the studies delineating the precise role of epigenetic alterations in inflammation and/or CVD in SS are just beginning, preliminary data suggest that pSS may represent an interesting model to study the factors involved in early development of AT.

## 4. miRNA Common to Different Autoimmune Diseases But Controlling Distinct Inflammatory Profiles: The Master Role of miR-146a

miR-146 and miR-155 have been shown to be induced by proinflammatory stimuli such as IL-1, TNF*α*, and Toll-like receptors (TLRs) [88]. They have also been detected in synovial fibroblasts, and rheumatoid PBMCs and synovial tissue, as well as in regulatory T cells serum, and urine cell free samples from SLE patients [89]. Both miRNAs have multiple targets, with miR-146 inhibiting TLR signalling and miR-155 regulating Th1 cells and also, interestingly, positively regulating mRNA for TNF*α*.

Concomitantly, Tang et al. [56] have shown that miR-146 regulates the level of at least TRAF6, IRAK1, STAT-1, and IFN regulatory factor 5 (IRF-5), all of which are important for the IFN pathway. The reported reduction of miR146 in PBMCs from SLE patients will likely affect the levels of these factors significantly and contribute to over expression of type I IFN and, in turn to disease activity.

Independent studies have demonstrated an increased level of miR146 in RA patients, but a decreased level in SLE patients, as compared with healthy controls. Given that RA and SLE are both systemic rheumatic diseases, one may be surprised by the finding that miR-146 levels are opposite in these diseases. Yet, as suggested by Chan et al. [90], this may simply reflect a difference in the overall cytokine profiles between the two diseases, with type I IFN playing a dominant role in SLE, whereas TNF*α*, interleukin-1, and IL-6 are the main cytokines in RA. That data reinforce the idea of the existence of some miRNAs as master gene regulators in different autoimmune diseases.

## 5. Control of DNA Methylation via miRNAs

Only recent studies have suggested that miRNAs can regulate DNA methylation by targeting the DNA methylation machinery in SLE. Pan et al. [91] identified 2 miRNAs, miR-21 and miR-148a, as being up-regulated in CD4+ T cells in both patients with lupus and MRL/lpr mice, an animal model of lupus. Moreover, both miRNAs down regulate the protein levels of DNMT1, thus resulting in hypomethylation status in CD4+ T cells. In particular, miR-21 indirectly down-regulates DNMT1 by targeting its upstream regulator, Ras guanyl-releasing protein 1, while miR-148a directly down regulates DNMT1 by targeting the protein-coding region of its transcript. The final result is the derepression of autoimmune-associated methylation-sensitive genes in CD4+ T cells, such as CD70 and lymphocyte function-associated antigen 1 (LFA-1; CD11a). These investigators were also able to induce the potential alleviation of hypomethylation in CD4+ T cells from patients with lupus by transfection with miR-21 and miR-148a inhibitors.

The study by Zhao et al. [92] further expanded the role of miRNAs and epigenetic changes in SLE. The novel finding was that, among the 11 microRNA that were observed to have increased or decreased expression in CD4+ T cells from patients with SLE, miR-126 was significantly over expressed, and its up-regulation was inversely correlated with DNMT1 protein levels. Zhao and colleagues were then able to demonstrate that miR-126 can directly inhibit DNMT1 translation by interacting with its 3′-UTR, leading to a significant reduction in DNMT1 protein levels. Through this mechanism, over expression of miR-126 causes demethylation and up-regulation of genes encoding for LFA-1 (CD11a) and CD70, two autoimmune-related proteins, which are directly proportional to disease activity. The miR-126 host gene EGFL7 was also over expressed in SLE CD4+ T cells, in a hypomethylation-dependent manner.

They were also able to show that knocking down miR-126 in SLE CD4+ T cells reduced their autoimmune activity and their stimulatory effect on IgG production in the cocultured B cells.

## 6. Therapeutic Potential of Epigenetic Modifications in Autoimmune Diseases

Unlike genetic alterations, which are permanent, epigenetic alterations are reversible. This opens up the possibility of using epigenetic drugs to reverse the pattern of epigenetic alterations to relieve the phenotype. To date, HDAC inhibitors such as suberoylanilide hydroxamic acid and trichostatin A (TSA) have proved to be useful for relieving lupus disease in mice [93]. The effects of TSA on human T cells are predominantly immunosuppressive and reminiscent of the signaling aberrations that have been described in patients with SLE.

Inhibition of HDACs has also been shown to alleviate renal disease in a mouse model of SLE [94, 95]. In line with this, it has been recently reported that HDAC inhibition is efficient in the treatment of juvenile idiopathic arthritis [96]. 

In autoimmune diseases, inhibiting DNA methylation would not be appropriate to revert DNA methylation changes, as the changes identified to date are hypomethylation, not hypermethylation; thus, agents should be designed to specifically increase methylation, and no such specific methylating agent exists. Nevertheless gene-gene specific hypermethylation cannot be ruled out [93].

Furthermore, DNA demethylating agents such as hydralazine have been shown to subvert B lymphocyte tolerance and to contribute to the generation of pathogenic auto reactivity [97].

It is not clear whether the increased expression of specific miRNAs is an indirect effect rather than the cause of SLE, and this point also needs further investigation in future studies. The hypothesis that many miRNAs are master regulators of gene expression remains attractive to select interesting target miRNAs for the development of new therapeutics.

## 7. Conclusions

There is a wealth of emerging evidence showing that epigenetics processes are involved in promoting autoimmunity. Yet, it remains unclear whether epigenetic changes in autoimmune diseases are causally related to the pathogenetic features, such as immune responses or inflammatory status, or whether they merely represent a consequence of the ongoing pathological process. However, epigenetic changes could at least partly explain poorly understood environmental effects on disease development and the enhanced cardiovascular risk observed in AIDs.

Epigenetic alterations can be used as clinical markers of disease progression or response to therapy. As stated above, hypomethylation, although very relevant to the pathology of the autoimmune disease, cannot be considered as a biomarker for development of alternative therapies. However, the current studies have indicated a huge potential of using miRNAs as gene therapy targets in vivo to treat cancer. In the same way, it can be anticipated that in the near future novel effective miRNA-based gene therapies will be developed to replace the traditional immune-suppressive therapies to treat autoimmune diseases. 

Identification of novel epigenetic targets, a better understanding of the epigenetic mechanisms involved in cardiovascular disease, and development of novel compounds directed against them will surely open up novel therapeutic approaches in systemic autoimmune diseases.
